# Supplementary figures and images for: MicroRNAs Expression in Response to rhNGF in Epithelial Corneal Cells: Focus on Neurotrophin Signaling Pathway
Source: Int J Mol Sci. 2022 Mar 25;23(7):3597. doi: 10.3390/ijms23073597 (PMC8998691; doi:10.3390/ijms23073597)

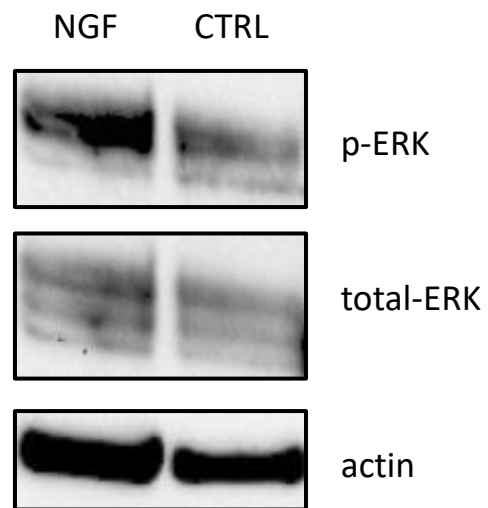

Figure S1: p-ERK induction after rhNGF treatment (30 min)

Supplement: Supplementary file 1 [file ijms-23-03597-s001.zip › ijms-1618192-SI/Supplementary Figure1.pdf]
